# Differential temporal decline of cerebral oxytocin and μ‐opioid receptor density during the aging process in mice

**DOI:** 10.1111/ejn.16578

**Published:** 2024-10-22

**Authors:** Felix Effah, Prakash Nidadavolu, Nívea Karla de Gusmão Taveiros Silva, Milosz Wojtowicz, Rosana Camarini, Andreas Zimmer, Alexis Bailey

**Affiliations:** ^1^ Pharmacology Section, St. George's School of Health & Medical Sciences City St George's University of London, Cranmer Terrace London UK; ^2^ Institute of Molecular Psychiatry, Medical Faculty, University of Bonn Bonn Germany; ^3^ Department of Neuroscience and Brain Technologies Istituto Italiano di Tecnologia, via Morego, 30 Genoa Italy; ^4^ Pharmacology Department Universidade de Sao Paulo São Paulo Brazil

**Keywords:** mu‐opioid receptor, neuroadaptations, ontogeny, oxytocin receptors

## Abstract

Aging is often associated with changes in social, sexual, emotional and pain functioning, as well as with the increased prevalence of certain psychopathologies. However, the neurodevelopmental basis underpinning these age‐related changes remains to be determined. Considering the key roles of oxytocin (OTR) and μ‐opioid (MOPr) receptor systems in regulating social, sexual, pain, reward and emotional processing, it seems plausible that they are also implicated in age‐related behavioural alterations. Although the ontogeny of both receptors has been well characterized in rodent brains from early development till adulthood, little is known concerning the neuroadaptations occurring from middle age to old age. Therefore, we mapped the neuroadaptations in OTR and MOPr in the brains of mice at those developmental endpoints. Quantitative OTR and MOPr autoradiographic binding was carried out in the brains of male mice at 2, 6, 9, 12 and 18 months of age. A significant whole brain decline in OTR density was detected between 2 and 6 months of age, with no additional decline thereafter. Interestingly, for MOPrs, the decline in density was not detected until 9 months of age. Region‐specific age‐related decline in OTR density was concentrated in the lateral anterior olfactory nuclei (AOL) and, for MOPr, in the AOL and the nucleus accumbens. Identifying the tipping point of these age‐related variations in both receptors may assist with our understanding of the neurobiology underlining age‐related changes in social, pain and emotional functioning/processing. It may also help us target interventions to specific developmental windows to abrogate certain age‐related psychopathologies.

## Graphical abstract

This study investigates the age‐dependent variations in Mu‐opioid receptor (MOPr) and oxytocin receptor (OTR) in mice from adolescence to geriatric age. The tipping point in OTR occurred at an earlier age than MOPR. This study may explain the onset of certain age‐related disorders mediated by OTR and MOPr.

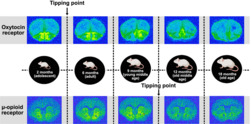

**Abbreviations**
AcbC: nucleus accumbens core
AcbSh: nucleus accumbens shell
AOV: anterior olfactory ventral
AOL: anterior olfactory lateral
AOM: anterior olfactory nucleus (anterior olfactory medial)
ASD: autistic spectrum disorder
AuD + AuV: auditory cortex
CA2 + CA3: CA2 and CA3 of the hippocampus
Cg1 + Cg2: anterior cingulate cortex
Den: dorsal endopiriform nucleus
KO: knockout
LO: lateral orbital cortex
LS: lateral septal nucleus
MO: medial orbital cortex
MOPr: Mu‐opioid receptor
MS: medial septal nucleus
OT: oxytocin
OTR: oxytocin receptor
Pir: piriform cortex
PND: postnatal day
S1: primary somatosensory cortex
Tu: olfactory tubercle
VDB: nucleus of the vertical limb of the diagonal band
TeA: temporal association cortex
V2L: visual cortex
VO: ventral orbital cortex

## 1 INTRODUCTION

Aging is a developmental process often associated with a decline in organ performance, including the brain. This can result in changes in motivation, stress regulation, pain tolerance, sexual behaviour and an increasing prevalence of certain neuropsychopathologies such as anxiety, depression and social withdrawal (Carstensen, 1992; Kay et al., 1985; Lindesay et al., 1989; Shoji et al., 2016). Similar manifestations can be observed in rodents, as numerous studies in rats and mice reported increased anxiety, depressive‐like behaviour, pain sensitivity and a decrease in social interactions, spatial and cued fear memory and analgesic opioid potency during the aging process (Boguszewski & Zagrodzka, 2002; Fahlström et al., 2012; Frick et al., 1999; Shoji et al., 2016; Sprott & Eleftheriou, 1974; Webster et al., 1976). The timing of the manifestation of those behavioural impairments depends on the age of the animals. For instance, a recent study by Shoji et al. (2016) detected decreases in locomotor activity in novel environments, motor function, acoustic startle response, social behaviour and an enhancement of depression‐related behaviour, increased prepulse inhibition and deficits in spatial and cued fear memory in the 4–5‐ and 6–7‐month‐old C57BL/6J mice compared to the 2–3‐month‐old group, with more pronounced changes in most behaviours observed in the 8–12 month‐old group. However, anxiety‐like behaviour increased significantly only in the older 8–12‐month‐old group, with no significant alterations in the younger groups. While those age‐related behavioural differences have been well established, the neurobiological processes underlying those changes are poorly understood. Understanding the nature of those aging‐related neuroadaptations is critical for the targeted intervention of age‐dependent neuropsychopathologies at appropriate developmental windows. Two potential neurobiological systems that may be implicated in the aforementioned age‐dependent behavioural decline based on their function are the oxytocin (OTR) and μ‐opioid receptor (MOPr) systems.

Oxytocin (OT), a hypothalamic neuropeptide, has been implicated in a broad spectrum of sexual, reproductive, emotional and social functioning in mammals (Caldwell et al., 1986; Lee et al., 2009; Tamma et al., 2009; Vaidyanathan & Hammock, 2017) and is critical for neuroplasticity. It plays a crucial role in sensory processing and social bonding through its interaction with the OTR (for review, see Muscatelli et al., 2018). The function of OT is exemplified by studies performed in OT or OTR knockout (KO) mice, which reveal deficits in social memory (Ferguson et al., 2001) and social interaction (Pobbe et al., 2012) and increased anxiety and stress responses to psychogenic and specific physiological stimuli (Amico et al., 2004; Mantella et al., 2003). Most of these behaviours were reversed by the administration of OT in OT‐deficient mice (Mantella et al., 2003), highlighting the prosocial properties of OT and its pivotal role in modulating a range of behaviours associated with stress‐related psychopathologies. Another neuropeptide receptor that is also recruited in response to natural rewards, including social rewards, and plays a crucial role in the modulation of pain, stress, reward, mood and well‐being is the MOPr (Le Merrer et al., 2009). Indeed, like in the case of OT/OTR KO mice, mice lacking MOPrs were recently proposed as a monogenic mouse model of autism spectrum disorder (ASD) (Becker et al., 2014; Pantouli et al., 2024; Pellissier et al., 2018), as they exhibited deficits in social behaviour, ASD‐like stereotyped and perseverative behaviours, as well as increased aggressiveness, exacerbated anxiety (Becker et al., 2014; Pantouli et al., 2024) and lowered nociceptive thresholds (Gavériaux‐Ruff & Kieffer, 2002; Pantouli et al., 2024).

Like most receptors, cerebral MOPr and OTR undergo profound ontogenic variations through development, especially during early development. In mice, evidence suggests the expression of MOPr mRNA as early as embryonic day 11 (Zhu et al., 1998). Homogenate binding studies in whole mouse brains revealed a 10% increase in MOPr binding density in postnatal (PND) 15 mice compared to PND3 mice and a further 25% increase at 10 weeks (Tavani et al., 1985). Age‐related differences in high‐affinity receptors labelled with MOPr agonist 3H‐dihydromorphine were determined using young (1‐month‐old), mature (3 and 8 months old) and aged (20 months old) mice (Ueno et al., 1988). MOPr binding sites in membranes from young and aged mice had significantly lower receptor densities than the mature mice. In a different mouse homogenate binding study using MOPr selective ligand [^3^H]DAMGO, MOPr binding density increased 2.7‐fold from PND 3 to PND7, with minor receptor density fluctuations evident from PND 7 to adulthood (Barg et al., 1992).

While MOPr ontogenic variations have been well characterized during early development, the impact of late aging on the MOPr system has been entirely neglected in mice and inconsistent in rat and humans. Age‐related increases (Gabilondo et al., 1995; Gross‐Isseroff et al., 1990; Kantonen et al., 2020; Zalsman et al., 2005; Zubieta et al., 1999), decreases (Fullerton et al., 2021; Kantonen et al., 2020; Piva et al., 1987; Ueno et al., 1988) and no change (Effah et al., 2022; Gulledge et al., 2000) in MOPr levels have been reported in rodent and human brains of older subjects in studies using radioligand binding or positron emission tomography. Interestingly, in old age, all PET or autoradiographic binding studies in the human brain detected increases in MOPr availability/binding in the neocortical regions, putamen or frontal and temporal cortical gyri (Gabilondo et al., 1995; Gross‐Isseroff et al., 1990; Zalsman et al., 2005; Zubieta et al., 1999). Consistent with those studies, a composite analysis of 11 PET studies using the MOPr selective radiotracer [11C] carfentanil in 132 healthy males spanning from 20 to 58 years of age detected an age‐related increase in MOPr availability in frontotemporal areas of the brain that appeared to plateau at 40–50 years of age (Kantonen et al., 2020). Interestingly, the same composite analysis (Kantonen et al., 2020) also revealed an age‐related reduction in MOPr availability in the thalamus, amygdala, nucleus accumbens and cerebellum in older age (Kantonen et al., 2020). Reduction in MOPr binding was also detected in the frontal poles, the striatum and the hippocampus of 24‐month‐old compared to 3‐month‐old rats (Hess et al., 1981) consistent with other rodent studies (Hess et al., 1981). Piva et al. (1987) also reported a decrease in the number of MOPr in the hypothalamus of 22‐month‐old rats compared to 2‐month‐old rats, and Fullerton et al. (2021) reported a similar decline in MOPr in the periaqueductal area (PAG) of 18‐month‐old rats compared to 2 months. Together, these findings suggest a differential impact of aging on different brain regions, with cortical regions showing upregulation and several non‐cortical regions associated with socio‐emotional processing (hypothalamus, amygdala, nucleus accumbens) and pain regulation (PAG, thalamus) showing a decline in old age.

OTR is present in mice and rats as early as the embryonic stage and increases during development (Tamborski et al., 2016; Yoshimura et al., 1996). Like in the case of MOPr, we found distinct ontogenic variations of OTR in different brain regions, with profound transient increases of OTR levels detected in specific olfactory nuclei, the septum and nucleus accumbens core of PND 22 rats compared to PND 8, which declined significantly in adulthood (PND 150) (Effah et al., 2021). Still, a steady decline from PND 8 to adulthood was detected in the cingulate cortex and caudate putamen in both male and female rats (Effah et al., 2021). However, no change in OTR density was detected in the motor, somatosensory and prelimbic cortex between PND 8, 22 and 150 (Effah et al., 2021). Similar patterns of ontogenic variations were detected by other groups in other rat strains and mice (Hammock & Levitt, 2013; Newmaster et al., 2020; Shapiro & Insel, 1989), suggesting that this pattern of OTR ontogenic variation is conserved among strains and species, at least in rodents. Interestingly, Newmaster et al. (2020) developed postnatal 3D template brains to register whole brain images with a cellular resolution to systematically quantify variations in temporal and spatial OTR cell densities using fluorescent reporter *Otr‐Venus* mice at five different postnatal days (PND 7, 14, 21, 28 and 56). Their data showed that overall cortical OTR density and olfactory areas peak at PND 21 and decrease into adulthood. In contrast, a steady increase in OTR cell density from PND 7 into adulthood was detected in hypothalamic and hippocampal areas in mice (Newmaster et al., 2020). However, using quantitative autoradiographic binding in male mice, Hammock and Levitt (2013) detected a peak in OTR binding at PND 14 in the neocortex and at PND 14 and PND 21 in the hippocampus, with a decline after that in young adulthood (PND 60).

Consistent with the MOPr situation, the handful of human and rat studies assessing the impact of aging on the OT system have been inconclusive while no studies have been carried out in mice. Some studies in rats (Fliers et al., 1985; Wierda et al., 1991; Zbuzek et al., 1988) did not detect any noticeable effect of aging on the OT fibre density, OT cell numbers or OT concentration in hypothalamic nuclei or plasma. Conversely, studies by Fliers and Swaab (1983), Zbuzek et al. (1988) and Ohno et al. (2018) reported age‐related changes like increased OT neurosecretory activity in the hypothalamus of 12‐, 18‐ and 24‐month aged rodents. Moreover, Elabd et al. (2014), Melis et al. (1992) and Ohno et al. (2018) reported decreased OT neurons in hypothalamic nuclei and/or reduction in plasma OT levels in aged rodents. In human post‐mortem brains, a reduction in the number of oxytocinergic cells in the hypothalamus of elderly (78 ± 2.3 year) subjects was reported (Calz et al., 1997). However, others found no changes in the number or size of oxytocinergic cells (Wierda et al., 1991). Concerning OTRs, only one study was published reporting a significant decrease in OTR binding in the caudate putamen, ventromedial hypothalamus and olfactory tubercle, but not in the anterior olfactory and amygdala nuclei of 20 months compared to 3‐month‐old rats (Arsenijevic et al., 1995).

It is still unclear what the behavioural consequences of these changes during aging in MOPr and OTR density are. However, given the vital role of OTR and MOPr systems in regulating social, sexual reward, pain and emotional modulation, these systems are likely to be implicated in those specific age‐related behavioural alterations (Onaka & Takayanagi, 2021).

Although the ontogeny of central MOPr and OTR have been well characterized in rodent brains from early development till adulthood, the spaciotemporal pattern of neuroadaptations from adolescence to middle age and old age is unknown, especially in mice. Therefore, this study aimed to unravel the ontogenic variations in OTR and MOPr systems from adolescence to adulthood to middle age to old age in 2‐, 6‐, 9‐, 12‐ and 18‐month‐old mice using quantitative receptor autoradiographic binding. To the best of our knowledge, this is the first study that directly and accurately dissected the impact of aging on the density of these receptors across multiple age groups in various brain regions in mice. Identifying the spatiotemporal pattern of potential age‐related variations in OTR and MOPr may assist with our understanding of the neurobiological underpinning of age‐related changes in social, sexual, pain and emotional functioning/processing. It may help us target treatment interventions for certain age‐related psychopathologies to specific time windows.

## 2 MATERIALS AND METHODS

### 2.1 Animals

Details of the animals used can be found in our recent publication (Nidadavolu et al., 2022). Briefly, male C57BL6/J mice (age groups: 2, 6‐, 9‐, 12‐ and 18‐month‐old mice) were purchased from Janvier, France. Before the brain samples were collected, mice were housed five in a cage and acclimatized for at least 2 weeks in our animal facility. All animal procedures were approved by the North Rhine‐Westphalia State Environment Agency (LANUV, Landesamt fuer Natur, Umwelt und Verbraucherschutz, license nr: AZ: 81–02.04.2019.A322) and were performed under the guidelines and the German Animal Protection Law regulating animal research.

### 2.2 Quantitative autoradiographic receptor binding

A total of 40 mice (*n* = 8 per age group) were sacrificed in accordance with the ARRIVE Guidelines 2.0, and whole brains were instantly frozen for 30 s in isopentane cooled below −20°C and immediately transferred to −80°C until later use. Serial coronal sections (20 μm thick) from Bregma 2.46 to −2.54 mm were sliced using a cryostat (‐21C) and mounted sequentially on two sets of pre‐labelled, 1% gelatin/chrome alum coated, Superfrost® Plus glass slides. The slides were carefully placed in a slide holder box kept on ice, then in an air‐tight container filled with Drierite™ with an indicator (Sigma‐Aldrich, Germany) at the base to keep the slides dry. The air‐tight box was then moved to 4°C for at least 2 h and then to −20°C for storage until use.

Quantitative autoradiographic receptor binding and film development were carried out according to the methods described in detail by Effah et al. (2022) and Georgiou et al. (2015, 2016). For OTR binding, two sets of slides having adjacent sections, one for total binding and the other for non‐specific binding, were used for the autoradiography experiment. As a preincubation step, the sections were rinsed for 10 min in a buffer solution (50 mM Tris–HCL pH 7.4, room temperature) to wash out endogenous OT. Slides were incubated for 60 min with 50 pM [^125^I]‐ornithine vasotocin analog [d (CH2)5[Tyr (Me)2,Thr4,Orn8,[125I]Tyr9‐NH2]‐vasotocin] ([^125^I]‐OVTA) (PerkinElmer, USA) in a buffer medium (50 mM Tris–HCl, 10 mM MgCl_2_, 1 mM EDTA, .1% w/v bovine serum albumin, .05% w/v bacitracin, pH 7.4 at room temperature) for the determination of total binding. For the nonspecific binding, adjacent sections were incubated with [^125^I]‐OVTA (50 pM) for 60 min in the presence of 50 μM of OT ligand (Thr4, Gly7)‐OT. Subsequently, slides were rinsed for 3 × 5 min in ice‐cold rinse buffer (50 mM Tris–HCl, 10 mM MgCl_2_, pH 7.4) followed by a 30‐min wash in the ice‐cold rinse buffer. Afterwards, the slides were washed for 2 s in ice‐cold distilled water.

For MOPr, the slides were preincubated in buffer solution (50 mM Tris–HCL pH 7.4, room temperature) for 30 min to wash out endogenous opioids. Slides were incubated for 60 min with 4 nM [^3^H]‐tyrosyl‐3,5‐3H (N) [D‐Ala 2, N‐MePhe 4, Gly‐ol]‐enkephalin (DAMGO) (PerkinElmer, USA) in a Tris–HCl buffer medium (pH 7.4, at room temperature) for the determination of total binding. For NSB, adjacent sections were incubated with 4 nM [^3^H]DAMGO in the presence 1 μM naloxone. Subsequently, the slides were rinsed 3 times for 5 min in ice‐cold (0°C) rinse buffer solution (50 mM Tris–HCl, 10 mM MgCl_2_, pH 7.4).

The slides used for MOPr and OTR underwent a 7‐day drying period while they were stored in sealed containers with anhydrous calcium sulphate (Drierite‐BDH Chemicals, Dorset, UK) They were then apposed to ^3^H‐ and ^125^I‐ sensitive film (Kodak MR‐1 films; Sigma‐Aldrich, UK) for 8 weeks for MOPr and 3 days for OTR together with ^3^H (for MOPr) and ^14^C (for OTR) microscale standards of known radioactive concentration. Film development was carried out in the dark under red‐filtered light. The films were developed in a 50% Kodak D19 developer solution (Sigma‐Aldrich, Poole, UK) for 3 min. The films were washed in distilled water containing drops of glacial acetic acid for 30 s, followed by 5 min in a Kodak rapid fix solution (Sigma‐Aldrich, Poole, UK). Finally, the films were rinsed in cold running water for 20 min and left to dry before being analyzed. The slides for either MOPr or OTR from all age groups were processed together regarding receptor binding, applied on the same film and processed together in a paired protocol.

### 2.3 MCID image analysis

Quantitative analysis of autoradiographic films was carried out by video‐based densitometry using the MCID computer software (Imaging Research, Canada), as previously described by us (Effah et al., 2021; Effah et al., 2022; Kitchen et al., 1997). For OTR, optical density values for 28 brain regions were quantified from the [^14^C]‐microscale standards of known radioactive concentration, cross‐calibrated with [^125^I] and then entered into a calibration table on MCID. For MOPr, optical density values for 31 brain regions were quantified from the [^3^H]‐microscale standards of known radioactive concentration and then entered into a calibration table on MCID. Specific binding was calculated by subtracting nonspecific binding from total binding and expressed as fmol/mg tissue equivalents. Brain structures were identified on the autoradiographic films by reference to the mouse atlas of Franklin and Paxinos (2019) at specific predetermined Bregma coordinates (see Supplementary Tables S1 & S2). The brain regions where the binding of OTR and MOPr was analyzed were selected based on previous autoradiographic binding analysis from our group (Effah et al., 2021, 2022; Georgiou et al., 2015, 2016).

### 2.4 Data analysis for quantitative receptor

Using GraphPad Prism, statistical analysis was conducted separately for MOPr and OTR binding. For MOPr and OTR binding, the mean specific binding ± standard error of the mean (*n* = 8) for each age in each brain region was determined. A two‐way analysis of variance (ANOVA) test was carried out for the factors ‘age’ and ‘region’ for each receptor binding, followed by Bonferroni post hoc tests where *p* < .05 was considered statistically significant. Planned comparisons using one‐way ANOVA (Bonferroni post hoc analyses) assessing age‐related changes in binding in all olfactory nuclei, the nucleus accumbens, the olfactory tubercle, piriform, dorsal endopiriform nucleus, medial septum, cingulate cortex, lateral septum, thalamus, hypothalamus, amygdala, ventral tegmental area and a periaqueductal grey area were carried out based on the a priori assumption that age‐related alterations in binding would occur in regions associated with social reward, mood, sexual behaviour, stress‐related emotional behaviour and pain regulation. Subsequently, all other brain regions were analysed by one‐way ANOVA. Box and whisker graphs with min to max were plotted, and statistical analysis was conducted using GraphPad Prism.

## 3 RESULTS

### 3.1 Ontogenic variation in OTR binding from young adulthood to old age

**FIGURE 1:**
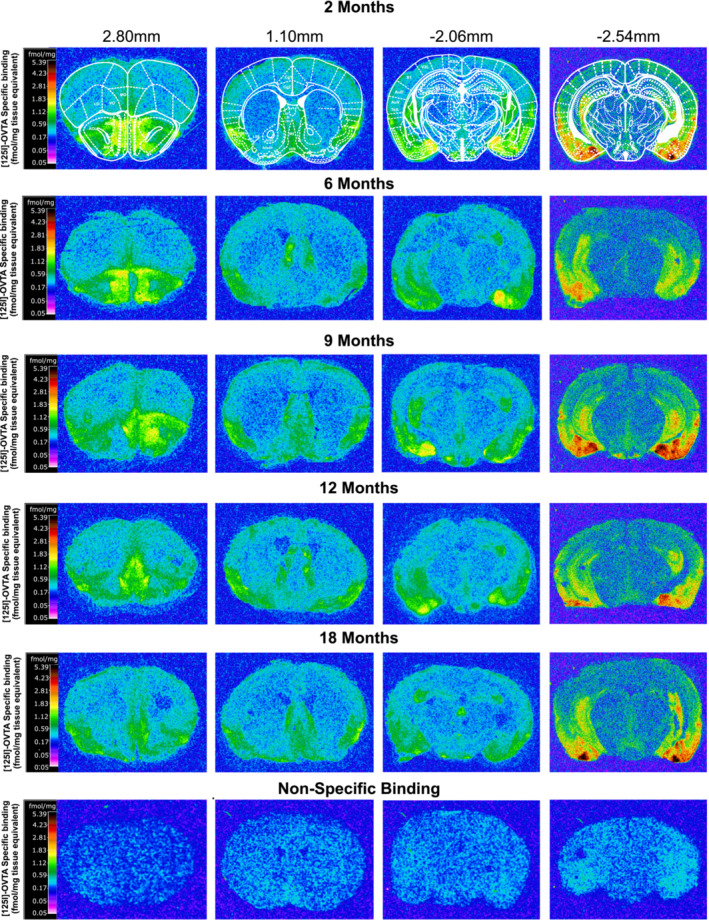
Computer‐enhanced representative autoradiograms of OTR binding in brains of 2‐, 6‐, 9‐, 12‐ and 18‐month‐old male C57BL6/J mice. The represented images are of total [125I]‐OVTA binding at the level of the anterior olfactory nucleus (Bregma 2.80 mm, first column), nucleus accumbens (Bregma 1.10 mm, second column) and thalamus and hypothalamus (−2.06 mm, third column), ventral tegmental area and periaqueductal grey (−2.54 mm, fourth column) of 2‐, 6‐, 9‐, 12‐ and 18‐month‐old mice. [125I]‐OVTA (50 pM) was used for total binding. Nonspecific binding images (bottom row) are from brain sections treated with [125I]‐OVTA (50pM) in the presence of 50 μM of (Thr4, Gly7)‐oxytocin. The colour bar illustrates a pseudocolor interpretation of black and white film images in fmol/mg tissue equivalent. Abbreviations of regions: AcbC + AcbSh, nucleus accumbens; anterior olfactory nucleus (AOL, anterior olfactory lateral; AOM, anterior olfactory medial; AOV, anterior olfactory ventral), AuD + AuV, auditory cortex; CA2 + CA3, CA2 and CA3 of the hippocampus; Cg1 + Cg2, anterior cingulate cortex; DEn, dorsal endopiriform nucleus; LO, lateral orbital cortex; LS, lateral septal nucleus; MO, medial orbital cortex; MS, medial septal nucleus; PAG, periaqueductal grey; Pir, piriform cortex, S1, primary somatosensory cortex; TeA, temporal association cortex; Tu, olfactory tubercle; V2L, visual cortex; VDB, nucleus of the vertical limb of the diagonal band; VO, ventral orbital cortex; VTA, ventral tegmental area.

Two‐way ANOVA showed a significant effect of age *F* (4, 940) = 9.301 (*p* < .0001) and region *F* (27, 940) = 152.5 (*p* < .0001), but no significant age × region interaction *F* (108, 940) = .9624 (*p* = .59). Bonferroni post‐hoc comparison between the 5 age groups across all regions revealed a significant age‐related decline in OTR binding in brains of 6‐ (*p* < .05), 9‐ (*p* < .01), 12‐ (*p* < .0001) and 18‐month (*p* < .0001)‐old mice compared to 2‐month‐old (Table 1). There was an 18% decline in OTR binding in the brains of 12‐ and 18‐month‐old mice compared to 2‐month‐old, and a 11–12% decline was detected in 6‐ and 9‐month‐old mice (Table 1). The tipping point for the decline was mostly observed between 2 and 6 months of age. Interestingly, no further significant decline was detected post 6 months of age (Table 1), suggesting that significant changes in OTR binding takes place only between 2 and 6 months. Representative autoradiograms of OTR binding in different age groups taken from 2.80, 1.10, −2.06 and −2.54 mm Bregma brain coordinates can be seen in Figure 1.

**TABLE 1:** Comparison of OTR binding between five age groups across all regions.

Age comparison	Mean 1 (fmol/mg)	Mean 2 (fmol/mg)	Mean diff.	% change	*t*	DF	Adjusted *p* value	Significance
2 months vs. 6 months	.6484	.5779	.07048	−11	3.240	940	.0124	*
2 months vs. 9 months	.6484	.5676	.08072	−12	3.596	940	.0034	**
2 months vs. 12 months	.6484	.5333	.1150	−18	5.287	940	<.0001	****
2 months vs. 18 months	.6484	.5318	.1166	−18	5.186	940	<.0001	****
6 months vs. 9 months	.5779	.5676	.01024	−2	.4429	940	>.9999	ns
6 months vs. 12 months	.5779	.5333	.04456	−8	2.071	940	.3859	ns
6 months vs 18 months	.5779	.5318	.04610	−8	2.002	940	.4559	ns
9 months vs. 12 months	.5676	.5333	.03433	−6	1.577	940	>.9999	ns
9 months vs. 18 months	.5676	.5318	.03586	−6	1.514	940	>.9999	ns
12 months vs. 18 months	.5676	.5318	.001539	0	.04881	940	>.9999	ns

Data represents the overall region of Bonferroni post‐hoc analysis following two‐way ANOVA.

**FIGURE 2:**
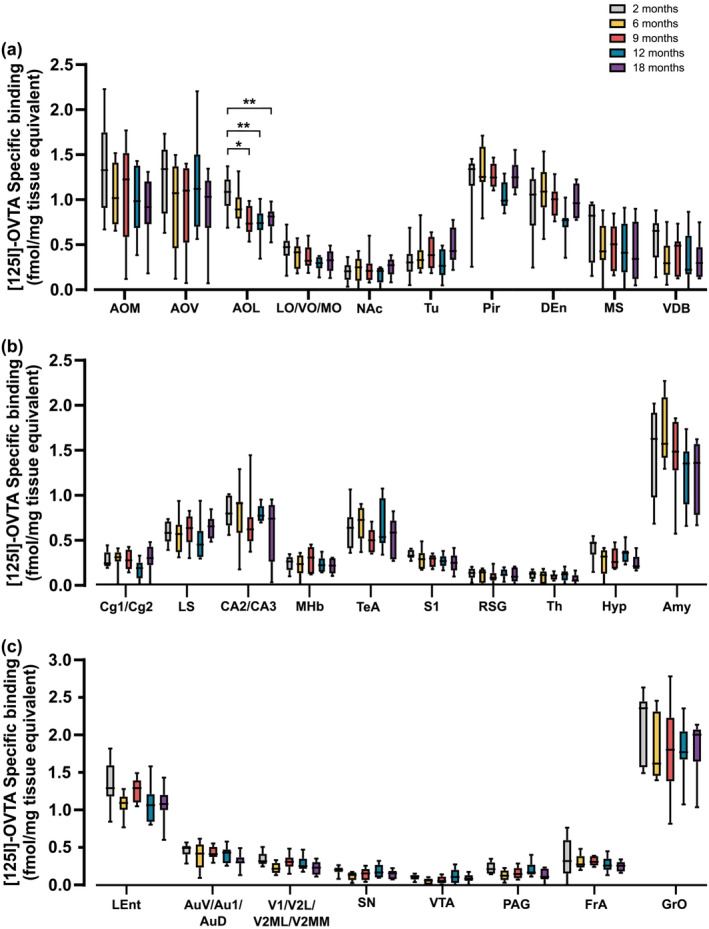
Age‐related changes in OTR density in brain regions of C57BL6/J mice. This figure illustrates [125I]‐OVTA‐specific binding in coronal brain sections of male mice at 2, 6, 9, 12 and 18 months of age. The [125I]‐OVTA concentration used for OTR labelling was 50 pM. Quantitative OTR binding levels are expressed as box and whiskers with min to max (*n* = 8 per group for most regions analyzed) specific [125I]‐OVTA binding (fmol/mg tissue equivalent). Specific binding was on average 70%–90% of total binding for higher expressing regions (>.9 fmol/mg total binding) and on average 30%–60% of total for lower expressions regions (<.9 fmol/mg total binding). *p < .05, **p < .01 (Bonferroni planned comparison analysis in all brain regions). Abbreviations of regions: Amy, amygdala; anterior olfactory nucleus (AOL, anterior olfactory lateral; AOM, anterior olfactory medial; AOV, anterior olfactory ventral); Au1 + AuD + AuV, auditory cortex; CA2 + CA3, CA2 and CA3 of the hippocampus; Cg1 + Cg2, anterior cingulate cortex; DEn, dorsal endopiriform nucleus; FrA, frontal association cortex; GrO, granule cell layer of the olfactory bulb; Hyp, hypothalamus; lent, lateral entorhinal cortex; LO, lateral orbital cortex; LS, lateral septal nucleus; MHb, medial habenular nucleus; MO, medial orbital cortex; MS, medial septal nucleus; NAc, nucleus accumbens; PAG, periaqueductal grey; Pir, piriform cortex; RSG, retrosplenial granular cortex; S1, primary somatosensory cortex; SN, substantia Nigra; TeA, temporal association cortex; Th, thalamus; Tu, olfactory tubercle; V1 + V2L + V2ML + V2MM, visual cortex; VDB, nucleus of the vertical limb of the diagonal band; VO, ventral orbital cortex; VTA, ventral tegmental area.

Based on the a priori assumption that age‐related binding alterations would occur specifically in regions associated with social reward, mood, sexual behaviour, stress‐related emotional behaviour and pain regulation, we carried out a planned comparison analysis to assess age‐related changes in binding in relevant brain regions. We detected a significant region‐specific decline in OTR binding as age advanced in 1 out of the 15 regions analysed: the lateral anterior olfactory nucleus (AOL) (Figure 2). A significant decline was seen in 9‐ (*p* < .05), 12‐ (*p* < .01) and 18‐month‐old mice AOL compared to 2‐month‐old. (Figure 2). No significant age‐related changes were detected in any of the other 27 regions analysed.

### 3.2 Ontogenic variation in MOPr binding from young adulthood to old age

**FIGURE 3:**
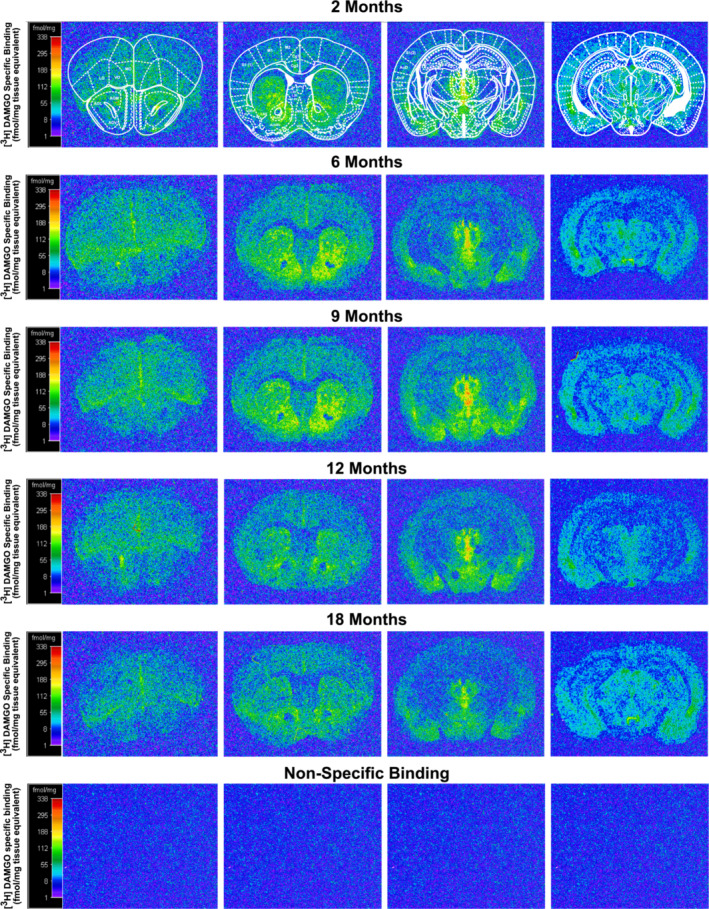
Computer‐enhanced representative autoradiograms of MOPr binding in the brain of 2‐, 6‐, 9‐, 12‐ and 18‐month‐old male C57BL6/J mice. The represented images are of total [3H]DAMGO binding of coronal brain sections at the level of the anterior olfactory nucleus (Bregma 2.80 mm, first column), nucleus accumbens (Bregma 1.10 mm, second column), thalamus and hypothalamus (Bregma −2.06 mm, third column), ventral tegmental area and periaqueductal grey (Bregma −2.54 mm, fourth column) of 2‐, 6‐, 9‐, 12‐, and 18‐month‐old mice. [3H]DAMGO (4 nM) was used for total binding. Non‐specific binding images (bottom row) were incubated with 4 nM [3H]DAMGO in the presence of 1 μM naloxone. The colour bar shows a pseudo‐colour interpretation of the relative density of black and white film images calibrated in fmol/mg tissue equivalent. Abbreviations of regions: anterior olfactory nucleus (AOL, anterior olfactory lateral; AOM, anterior olfactory medial; AOV, anterior olfactory ventral); AuD + AuV, auditory cortex; CA2 + CA3, CA2 and CA3 of the hippocampus; Cg1 + Cg2, anterior cingulate cortex; DEn, dorsal endopiriform nucleus; LO, lateral orbital cortex; LS, lateral septal nucleus; M1 + M2, primary and secondary motor cortex; MHb, medial Habenular nucleus; MO, medial orbital cortex; MS, medial septal nucleus; nucleus accumbens (nucleus accumbens core + nucleus accumbens shell [NAc]); PAG, periaqueductal grey Pir, piriform cortex; S1, primary somatosensory cortex; S2, secondary somatosensory cortex; TeA, temporal association cortex; Tu, olfactory tubercle; VDB, nucleus of the vertical limb of the diagonal band; VO, ventral orbital cortex; VTA, ventral tegmental area.

Two‐way ANOVA showed a significant effect of age *F* (4, 1011) = 14.02 (*p* < .0001) and region *F* (30, 1011) = 24.04 (*p* < .0001), but no significant age × region interaction *F* (120, 1011) = .7121 (*p* = .9). Bonferroni post‐hoc comparison between the 5 age groups across all regions revealed a significant age‐related decline in MOPr binding in the brains of 12‐ and 18‐month‐old vs 2‐ and 6‐month‐old mice (*p* < .05), and in the brains of 18‐month‐old vs 9‐month‐old mice (*p* < .001) (Table 2). However, no significant decline was detected in 6‐ and 9‐month‐old mice compared to 2‐month‐old mice (Table 2), suggesting that the decline in MOPr density does not occur until middle age (after 9 months of age) with a 12–19% decline in MOPr detected in 12‐ and 18‐month‐old mice, when compared to 2‐ and 6‐month‐old mice (Table 2). Representative autoradiograms of MOPr binding in different age groups taken from 2.80, 1.10, −2.06 and −2.54 mm Bregma brain coordinates can be seen in Figure 3.

**TABLE 2:** Comparison of MOPr binding between five age groups across all regions.

Age comparison	Mean 1 (fmol/mg)	Mean 2 (fmol/mg)	Mean diff.	% change	t	DF	Adjusted *p* value	Significance
2 months vs. 6 months	46.29	44.63	1.658	−4	1.274	1011	>.9999	ns
2 months vs. 9 months	46.29	43.12	3.165	−7	2.394	1011	.1684	ns
2 months vs. 12 months	46.29	40.72	5.564	−12	4.289	1011	.0002	***
2 months vs. 18 months	46.29	37.38	8.906	−19	6.805	1011	<.0001	****
6 months vs. 9 months	44.63	43.12	1.508	−3	1.122	1011	>.9999	ns
6 months vs. 12 months	44.63	40.72	3.906	−9	2.962	1011	.0313	*
6 months vs 18 months	44.63	37.38	7.248	−16	5.449	1011	<.0001	****
9 months vs. 12 months	43.12	40.72	2.399	−6	1.791	1011	.7356	ns
9 months vs. 18 months	43.12	37.38	5.74	−13	4.251	1011	.0002	***
12 months vs. 18 months	40.72	37.38	3.342	−8	2.52	1011	.1187	ns

Data represents the overall region of Bonferroni post‐hoc analysis following two‐way ANOVA.

**FIGURE 4:**
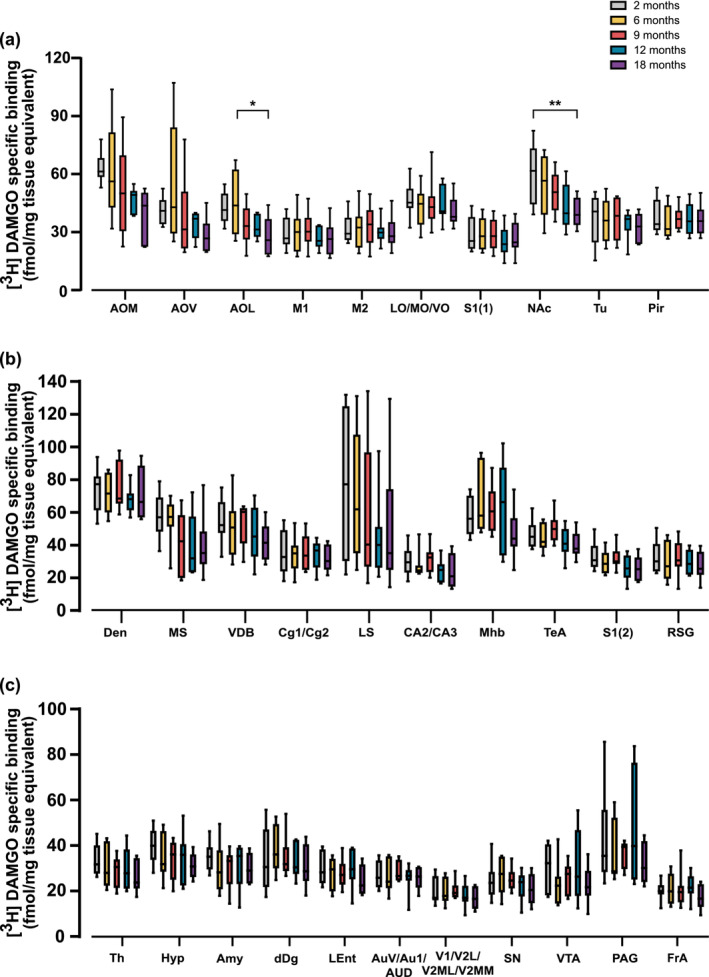
Age‐related changes in MOPr density in brain regions of male C57BL6/J mice. This figure illustrates [3H] DAMGO‐specific binding in coronal brain sections from male mice at 2, 6, 9, 12 and 18 months of age. The concentration of [3H]DAMGO used for MOPr labelling was 4 nM. Data are expressed as box and whiskers with min to max (*n* = 8 per group for most regions analyzed) [3H]DAMGO specific binding (fmol/mg tissue equivalent). Specific binding was 100% of total binding for all regions analyzed. *p < .05, **p < .01 (Bonferroni planned comparison analysis in all brain regions). Abbreviations of regions: Amy, amygdala; anterior olfactory nucleus (AOM, anterior olfactory medial; AOV, anterior olfactory ventral; AOL, anterior olfactory lateral); Au1 + AuD + AuV, auditory cortex; CA2 + CA3, CA2 and CA3 of the hippocampus; Cg1 + Cg2, anterior cingulate cortex; dDG, dorsal dentate gyrus; DEn, dorsal endopiriform nucleus; FrA, frontal association cortex; lent, lateral entorhinal cortex; LO, lateral orbital cortex; Hyp, hypothalamus; LS, lateral septal nucleus; M1 + M2, primary and secondary motor cortex; MHb, medial habenular nucleus; MO, medial orbital cortex; MS, medial septal nucleus; PAG, periaqueductal grey; S1, primary somatosensory cortex; nucleus accumbens (nucleus accumbens core + nucleus accumbens shell [NAc]); Pir, piriform cortex; RSG, retrosplenial granular cortex; S1, primary somatosensory cortex; SN, substantia Nigra; TeA, temporal association cortex; Th, thalamus; Tu, olfactory tubercle; V1 + V2L + V2ML + V2MM, visual cortex; VDB, nucleus of the vertical limb of the diagonal band; VO, ventral orbital cortex; VTA, ventral tegmental area.

Based on the a priori assumption that age‐related binding alterations would occur specifically in regions associated with social reward, mood, sexual behaviour, stress‐related emotional behaviour and pain regulation, we carried out a planned comparison analysis to assess age‐related changes in binding in relevant brain regions. We detected a significant region‐specific decline with age in 2 of the 31 regions analyzed; AOL and the nucleus accumbens (NAc), where a significant decrease was detected in 18‐month‐old vs 6‐month‐old mice, and in 18‐month‐old vs 2‐month‐old mice, respectively (Figure 4).

## 4 DISCUSSION

In the present study, we characterized the ontogenic variations in the OTR and MOPr systems in the brains of mice ranging from adolescence to old age in five distinct age groups. We followed the age‐categorization of Jackson Laboratory and other studies (https://www.jax.org/news-and-insights/jax-blog/2017/november/when-are-mice-considered-old [Fox, 2007; Jackson et al., 2017]); therefore, 2‐month‐old mice were termed as an adolescent, 6‐month‐old as an adult, 9‐ and 12‐month‐old as middle‐aged and 18‐month‐old as old. Our findings revealed a significant generalized overall region and region‐specific age‐related decrease in the density of OTR and MOPr in the mouse brain. Interestingly, the temporal tipping point for this decline differed among the two receptor systems, with an overall OTR decrease taking place during young adulthood (between 2 and 6 months of age) and no further decline beyond that, and the decline in MOPr occurring during the middle age (after 9 months).

The whole brain decline in OTR density from 2 (adolescence) to 6 months (adult) detected in this study is broadly consistent with our recent findings in rats (Effah et al., 2021) and others (Hammock & Levitt, 2013; Newmaster et al., 2020) in mice, who also found a decline in OTR density in adulthood after an upregulation in PND 22 (weaning age) in specific brain regions. This observation is also broadly consistent with a recent study by Rokicki et al. (2021), who used genome‐wide exon‐level transcriptome data from 57 human donors across 16 brain regions to show a decline in OTR gene expression in adulthood after an increase in early childhood (19 months) (Audunsdottir & Quintana, 2022). The evidence clearly demonstrates the conservation of these ontogenic plastic changes in OTR among different species, at least during early development until adulthood.

In agreement with Arsenijevic et al. (1995), who compared 20 months with 3‐month‐old rats, we detected an 18% overall decrease in OTR binding in the brains of 18‐month‐old mice compared to 2‐month‐old, suggesting that, like in early development, ontogenic plastic changes in OTR may be conserved among species, at least in rodents. The major finding of the present study, however, rests on the determination that this decline took place in adulthood, between 2 and 6 months of age in mice, with no further additional decline in middle age or even in old age. This finding suggests that the turning point for the decline in OTR is concentrated in adulthood. In contrast with the current study, based on genome‐wide exon‐level transcriptome data from 57 human donors across 16 brain regions, Rokicki et al. (2021) detected increased OTR expression in late adulthood, which was especially pronounced in the cerebellar cortex. While this data may reflect species differences in OTR patterns in old age, the discrepancy in findings between the studies may also be due to differences in analytical techniques used to measure OTR. Indeed, Rokicki et al. (2021) used transcriptome data as a proxy for gene expression density in males and females. In contrast, we used receptor autoradiographic binding to measure male OTR protein density, which is a far more direct methodological approach.

The cause for the age‐related decline in OTR binding is unclear. Still, several factors may be involved: (1) Firstly, one proposal would be that it may be triggered by fluctuations in the circulating gonadal steroids during aging. Indeed, gonadal steroids (oestrogen and testosterone) are known to regulate OTR density in rodent brains (Arsenijevic & Tribollet, 1998) and given the age‐related decline of testosterone post‐young adulthood in males (McBride et al., 2016); it is conceivable that changes in testosterone levels through aging may at least be partly responsible for the observed decrease in OTR's between 2‐ and 6‐month‐old mice in the present study. However, if that was the case, one would have expected a linear age‐related decline in OTR, as testosterone levels steadily decrease post‐young adulthood with the lowest circulating levels present at old age (McBride et al., 2016), which is something not detected in our study. (2) An alternative explanation could be that the OTR decline may be related to the significant decrease in striatal dopamine content (Algeri et al., 1977; Demarest et al., 1980), dopamine release (Yurek et al., 1998) and D2 dopamine receptors (Zoli et al., 1990) during aging in rodents. Given the well‐characterised OT interaction with the dopamine system, which is implicated in regulating socioemotional and sexual behaviour (Baskerville & Douglas, 2010), it is conceivable that this age‐related OTR decline may be related to the age‐related changes in the dopaminergic system. In support of this, Rokicki et al. (2021) detected a strong spatiotemporal correlation between OTR and D2 gene expression in the brains of human donors throughout the lifespan, thus suggesting that the OT system works synergistically with the dopaminergic signalling throughout development to support socioemotional wellbeing. (3) This decline may be a compensatory neuroadaptation to age‐related increases in endogenous OT release in blood and/or brain. However, as discussed in the introduction, the literature so far has yielded inconclusive results in that respect, although Fliers and Swaab (1983), Zbuzek et al. (1988) and (Ohno et al., 2018) detected an increase in OT neurosecretory activity from the hypothalamus in old age. More research is needed to clarify the situation.

Interestingly, planned comparison in selected regions associated with social functioning, emotional regulation and sexual behaviour revealed significant OTR alterations only in the OTR‐rich lateral olfactory nuclei (AOL), with a significant decline taking place in 9‐month‐old mice compared to 2 months, with no further decline thereafter. A recent study using optogenetic technology in rats demonstrated the crucial role of the anterior olfactory nucleus (AON) in olfactory sensory processing required for social interactions, with selective deletion of AON OTR triggering impairment in social recognition (Oettl et al., 2016). Whether the deficits in OTR in the AOL contribute to the age‐related decline in social recognition and processing remains to be determined.

Understanding OTR changes during the aging process is particularly important to shed light on the molecular mechanisms underlying the age‐related socioemotional adaptations, which may have implications in determining the efficacy and therapeutic timeframe of OT‐based interventions. The therapeutic potential of intranasal OT in improving socioemotional functioning has been well documented in multiple preclinical and human studies reviewed by Ebner et al. (2013). However, most of those studies were conducted in adolescents and adults, with very few in older individuals. While Keck et al. (2000) reported blunted OT responses to stress in old rats, the effects of exogenously administered OT on social memory and depressive‐like behaviour seem to be preserved in aging rats (Arletti et al., 1995). The results of 10‐day randomized control trials focusing on OT's effect on social engagement in old men (Barraza et al., 2013) suggested an improvement in dispositional gratitude in the OT group compared to placebo with no change in mood, social activity or engagement patterns across the study interval. More comparative studies focusing on OT's effect on social functions between different age groups (notable young adult, adult, middle age, and old age) are warranted to ascertain the impact of aging on the efficacy of intranasal OT on socioemotional functioning and to determine the age window where OT may have its highest efficacy. For instance, OT may exert higher therapeutic potential when administered during an aging timeframe characterized by the decline of the oxytocinergic system; a hypothesis also put forward by Ebner et al. (2013).

Consistent with the OTR temporal pattern of variation during the aging process, we also detected an overall age‐related non‐linear decline in MOPr density in mice brains. However, in contrast with OTRs, the decline in MOPr binding occurred later on, after 9 months of age (i.e. during middle age), with no further decline after that, clearly suggesting the existence of distinct but differential patterns of temporal decline of MOPr and OTR density during the aging process. Interestingly, we recently demonstrated, using the same age groups and strain of mice, that the ontogenic variations of the endogenous endocannabinoid system, including CB1 receptors, are also concentrated in the middle‐age (Nidadavolu et al., 2022).

The lack of changes in MOPr density from 2 (adolescence) to 6 months (adulthood) detected in this study is largely consistent with our recent study in rats (Effah et al., 2022), where we were also unable to detect any changes in MOPr density post PND 22 until adulthood in both males and females. Nonetheless, this contrasts with a recent comparative study by Smith et al. (2018), who found a significant decline in MOPr in 11 out of 33 brain regions of 3‐month‐old (adult) compared to juvenile (1‐month‐old) rats. The lack of consistency between our studies could be due to species differences (mice vs rats), differences in age groups (1 vs 2 months and 3 vs 6 months) and/or differences in environmental conditions animals were reared in, or a combination of those.

The region‐specific decline in MOPr in old age reported in the literature in rodent and human brains (Fullerton et al., 2021; Hess et al., 1981; Kantonen et al., 2020; Piva et al., 1987; Ueno et al., 1988) is consistent with the current study, which detected a 12%–19% overall region decrease in MOPr in old age mice (12–18 months) compared to adults (2–6 months) with prominent region‐specific age‐related decline in the AON and the nucleus accumbens. The significant finding of the current study rests towards the temporal identification that the decline does not occur until middle age in mice and rats, with no further decline in old age, suggesting a lack of linear decline of MOPr with old age (Fullerton et al., 2021), which is not rodent‐specific. Still, that decline is concentrated during the middle ages.

Like in the case of OTR, several factors may drive the age‐related MOPr decline. (1) This could be compensatory neuroadaptation due to the high secretion of endogenous opioids (endorphins and enkephalins) (Lesscher et al., 2003) in the aging brain. There are conflicting reports concerning the direction of changes in endogenous opioids in the aging brain, with studies reporting increases (Kumar et al., 1980) and others reporting decreases (Sonntag et al., 1980) in endogenous opioid concentration in rodent CNS. It is thus difficult to make any definite conclusion in that respect. (2) Alternatively, the reduction in MOPr binding may reflect changes in MOPr affinity rather than density. However, saturation binding studies in rodents confirmed that the changes in MOPr binding also reflected changes in Bmax rather than Kd (Barg et al., 1992; McDowell & Kitchen, 1988; Spain et al., 1985; Tavani et al., 1985; Zagon, 1998) thus rendering this possibility unlikely. (3) It could be partly caused by brain atrophy during the late aging process. Grey matter is reduced during aging (Lockhart & DeCarli, 2014; Taylor et al., 2020), which is concomitant, as discussed above, with loss of dopamine content (Algeri et al., 1977; Noda et al., 2020), dopamine release (Demarest et al., 1980) and D2 dopamine receptors (Zoli et al., 1990), which takes place with advancing age. This possibility is plausible given the close interaction between the dopamine and endogenous MOPr system, especially in the nucleus accumbens (Le Merrer et al., 2009).

The behavioural consequence of this age‐related decline in MOPr remains to be elucidated. However, given the role of MOPr in the modulation of pain processing, MOP‐mediated analgesia, reward and emotional regulation, it is plausible that this decline may at least be partly involved in the decline in pain processes, opioid analgesic potency, socioemotional and reward processing during old age. Several studies have reported a decrease in pain sensitivity with old age (Lautenbacher et al., 2017) and a reduction in MOPr‐mediated analgesic potency with aging, such that aged male rats required a significantly higher MOPr agonist dosage to reach analgesia than adult rats (Fullerton et al., 2021). Hence, although the age‐related decline in MOPr is unlikely to explain the changes in pain threshold during the aging process, as pain sensitivity decreases in old age (Lautenbacher et al., 2017), it is however likely at least to explain the age‐related changes in opioid analgesic potency.

Interestingly, planned comparison in our study singled out the nucleus accumbens (NAc) as one of the two brain regions where the age‐related decline in MOPr reached significance. The NAc is a critical region mediating motivational, rewarding and emotional processes. Impairment of this structure has been implicated in numerous CNS and psychiatric disorders, including depression, obsessive‐compulsive disorder, bipolar disorder, anxiety disorders, Parkinson's disease, Alzheimer's disease, Huntington's disease, obesity, drug abuse and addiction (Salgado & Kaplitt, 2015). MOPr is highly expressed in the NAc, and stimulation of these receptors in this structure has been shown to play a key role in mediating hedonic properties of natural (social, sex, food etc.) and drug rewards with impairment in NAc MOPr signalling, suppressing some of those reinforcing behaviour (Le Merrer et al., 2009; Trezza et al., 2011). As such, this age‐related decline in MOPr detected in the NAc in our study could be implicated in the decline of socioemotional functioning and anhedonia in old age. Further research is warranted to test this hypothesis. Moreover, it is conceivable that the detected MOPr decline in the nucleus accumbens in old age mice may reflect age‐related changes in central pain processing, as fibromyalgia, which is a condition characterised by altered pain processing, is associated with reduced opioid receptor availability in the nucleus accumbens, among other brain regions (Harris et al., 2007). Taking all the above together and the fact that the tipping point for MOPr decline is concentrated in middle age, our findings may have implications on how pain is managed with opioids at this time frame and highlights the optimal time window for potential opioid‐mediated therapeutic interventions to abrogate the course of socioemotional and/or painful aging.

Interestingly and consistent with the significant age‐related decline in OTR in that region detected in this study, we also detected a significant age‐related decline in MOP in the AOL. Whether the decline in both receptors in the same region reflects an interplay between these receptors in that brain region, as suggested in our previous study (Georgiou et al., 2015), remains to be determined. Nonetheless, this study suggests that changes in the density or functioning of OTR and MOPr in various brain regions do not follow a uniform pattern or occur simultaneously during the mouse's lifespan. Instead, these declines appear to happen at distinct time points, suggesting that the regulation or adaptation of these receptor systems may be influenced by different factors or mechanisms at different stages of the mouse's life.

As cognitive decline is a cardinal symptom of old age and given the pivotal role of OTR and MOPr in cognition (Jafari‐Sabet & Jannat‐Dastjerdi, 2009), we assessed the impact of aging on MOPr and OTR binding in the CA2/CA3 hippocampal region, which serves a critical function in memory. Nonetheless, we observed no significant age‐dependent changes in either of these receptors in that region. Hence, if MOPr and OTR decline are involved in age‐related cognitive decline, it is due to mechanisms other than receptor decline in the hippocampus.

A limitation of this study is the exclusive use of male mice, which restricts the generalizability of the findings, mainly because of the sex‐dependent MOPr and OTR effects. While sex differences in OTR binding during embryonic and early development have been identified (Hammock & Levitt, 2013; Newmaster et al., 2020; Tamborski et al., 2016), it is unclear whether these exist in old age. The study by Hammock and Levitt (2013) shows that sex differences disappear in adulthood, at least in the neocortex, septum and hippocampus. In contrast to OTR, there have been limited sex differences detected in MOPr binding at early developmental stages until adulthood, with the vast majority of brain regions showing no change in MOPr binding in females (Effah et al., 2022; Smith et al., 2018). Future studies, including female mice, should investigate the presence or absence of sex‐dependent ontogenic variations. This could provide critical insights into the role of sex hormones, which may be necessary in understanding the sex‐related prevalence of certain age‐related disorders mediated by OTR and MOPr.

## 5 CONCLUSION

In conclusion, we demonstrated for the first time a distinct but differential temporal decline of cerebral OTR and MOPr density during the aging process, with OTR decline occurring as early as adulthood and MOPr taking place in middle age, with no further decline thereafter. Identifying the tipping point of these age‐related variations in OTR and MOPr may not only assist us with our understanding of age‐related changes in social, sexual, pain and emotional functioning and processing, but it may also help us target treatment interventions to specific time windows to abrogate socioemotional and/or pain‐related consequences of aging.

## AUTHOR CONTRIBUTION

Felix Effah: data curation, formal analysis, investigation, visualization, writing—original draft, writing—review and editing. Prakash Nidadavolu: conceptualization, data curation, formal analysis, investigation, writing—review and editing. Nivea Karla de Gusmão Taveiros Silva: formal analysis, investigation, visualization, writing—review and editing. Milosz Wojtowicz: data curation, writing—review and editing. Rosana Camarini: writing—review and editing. Andreas Zimmer: conceptualization, resources, writing—original draft, writing—review and editing. Alexis Bailey: conceptualization, resources, formal analysis, writing—original draft, writing—review and editing.

## CONFLICT OF INTEREST STATEMENT

The authors declare no conflicts of interest.

### PEER REVIEW

The peer review history for this article is available at https://www.webofscience.com/api/gateway/wos/peer-review/10.1111/ejn.16578.

Abbreviations: ANOVA, analysis of variance; ns, no significance; OTR, oxytocin receptor.

Abbreviations: ANOVA, analysis of variance; MOPr, Mu‐opioid receptor; ns, no significance.

## Supporting information


**Table S1.** The brain regions analyzed for OTR binding with the Bregma coordinates from which they were identified.
**Table S2**. The brain regions analyzed for OTR binding with the Bregma coordinates from which they were identified.

## Data Availability

All data are included in this submission.
