# Supplementary material for: Differential temporal decline of cerebral oxytocin and μ‐opioid receptor density during the aging process in mice
Source: Eur J Neurosci. 2024 Oct 22;60(11):6686–703. doi: 10.1111/ejn.16578 (PMC11612844; doi:10.1111/ejn.16578)
Supplement: Supplementary file 1 — Table S1. The brain regions analyzed for OTR binding with the Bregma coordinates from which they were identified. Table S2. The brain regions analyzed for OTR binding with the Bregma coordinates from which they were identified. [file EJN-60-6686-s001.docx]

**Supplementary Table 1. The brain regions analyzed for OTR binding with the Bregma coordinates from which they were identified.**

| OTR Binding | |
| --- | --- |
| Bregma Coordinates | **Brain Region Analysed** |
| 2.80mm | Anterior Olfactory Nucleus (AOM + AOV + AOL)  Lateral Orbital Cortex (LO)  Ventral Orbital Cortex (VO)  Medial Orbital Cortex (MO)  Frontal Association Cortex (FrA)  Granule Cell Layer of the Olfactory Bulb (GrO) |
| 1.10mm | Nucleus Accumbens (AcbC + AcbSh)  Olfactory Tubercle (Tu)  Piriform Cortex (Pir)  Dorsal Endopiriform Nucleus (DEn)  Medial Septal Nucleus (MS)  Nucleus of the vertical limb of the diagonal band (VDB)  Anterior Cingulate Cortex (Cg1 + Cg2)  Lateral Septal Nucleus (LS) |
| -2.06mm | Fields CA2 and CA3 of the Hippocampus (CA2 + CA3)  Medial Habenular nucleus (MHb)  Temporal Association Cortex (TeA)  Primary Somatosensory Cortex (S1)  Retrosplenial Granular Cortex (RSG)  Thalamus (Th)  Hypothalamus (Hyp)  Amygdala (Amy) |
| -2.54mm | Lateral Entorhinal Cortex (LEnt)  Auditory Cortex: (Au1 + AuD + AuV)  Visual Cortex: (V1 + V2L + V2ML + V2MM)  Substantia Nigra (SN)  Ventral Tegmental Area (VTA)  Periaqueductal Grey (PAG) |

**Supplementary Table 2. The brain regions analyzed for OTR binding with the Bregma coordinates from which they were identified.**

| MOPr Binding | |
| --- | --- |
| Bregma Coordinates | **Brain Region** |
| 2.80mm | Anterior Olfactory Nucleus (AOM + AOV + AOL)  Lateral Orbital Cortex (LO)  Ventral Orbital Cortex (VO)  Medial Orbital Cortex (MO)  Frontal Association Cortex (FrA) |
| 2.10mm | Primary and Secondary Motor Cortex (M1 + M2) |
| 1.98mm | Primary Somatosensory Cortex (S1) |
| 1.10mm | Nucleus Accumbens (AcbC + AcbSh)  Olfactory Tubercle (Tu)  Piriform Cortex (Pir)  Dorsal Endopiriform Nucleus (DEn)  Medial Septal Nucleus (MS)  Nucleus of the vertical limb of the diagonal band (VDB)  Anterior Cingulate Cortex (Cg1 + Cg2)  Lateral Septal Nucleus (LS) |
| -2.06mm | Fields CA2 and CA3 of the Hippocampus (CA2 + CA3)  Medial Habenular nucleus (MHb)  Temporal Association Cortex (TeA)  Primary Somatosensory Cortex (S1)  Retrosplenial Granular Cortex (RSG)  Thalamus (Th)  Hypothalamus (Hyp)  Amygdala (Amy)  Dorsal Dentate Gyrus (dDG) |
| -2.54mm | Lateral Entorhinal Cortex (LEnt)  Auditory Cortex: (Au1 + AuD + AuV)  Visual Cortex: (V1 + V2L + V2ML + V2MM)  Substantia Nigra (SN)  Ventral Tegmental Area (VTA)  Periaqueductal Grey (PAG) |
